# Personalised Hip Therapy: development of a non-operative protocol to treat femoroacetabular impingement syndrome in the FASHIoN randomised controlled trial

**DOI:** 10.1136/bjsports-2016-096368

**Published:** 2016-10

**Authors:** Peter DH Wall, Edward J Dickenson, David Robinson, Ivor Hughes, Alba Realpe, Rachel Hobson, Damian R Griffin, Nadine E Foster

**Affiliations:** 1 Warwick Medical School, University of Warwick, Coventry, UK; 2 Southbank Hospital Worcester, Spire Healthcare, Worcester, UK; 3 University Hospitals Coventry and Warwickshire, Coventry, UK; 4 University Hospitals of Coventry and Warwickshire NHS Trust and Warwick Medical School, University of Warwick, Coventry, UK; 5 Arthritis Research UK Primary Care Centre, Research Institute of Primary Care and Health Sciences NIHR Professor of Musculoskeletal Health in Primary Care, Keele University, Keele, UK

**Keywords:** Hip, Exercise rehabilitation, Physiotherapy, Orthopaedics

## Abstract

**Introduction:**

Femoroacetabular impingement (FAI) syndrome is increasingly recognised as a cause of hip pain. As part of the design of a randomised controlled trial (RCT) of arthroscopic surgery for FAI syndrome, we developed a protocol for non-operative care and evaluated its feasibility.

**Methods:**

In phase one, we developed a protocol for non-operative care for FAI in the UK National Health Service (NHS), through a process of systematic review and consensus gathering. In phase two, the protocol was tested in an internal pilot RCT for protocol adherence and adverse events.

**Results:**

The final protocol, called Personalised Hip Therapy (PHT), consists of four core components led by physiotherapists: detailed patient assessment, education and advice, help with pain relief and an exercise-based programme that is individualised, supervised and progressed over time. PHT is delivered over 12–26 weeks in 6–10 physiotherapist-patient contacts, supplemented by a home exercise programme. In the pilot RCT, 42 patients were recruited and 21 randomised to PHT. Review of treatment case report forms, completed by physiotherapists, showed that 13 patients (62%) received treatment that had closely followed the PHT protocol. 13 patients reported some muscle soreness at 6 weeks, but there were no serious adverse events.

**Conclusion:**

PHT provides a structure for the non-operative care of FAI and offers guidance to clinicians and researchers in an evolving area with limited evidence. PHT was deliverable within the National Health Service, is safe, and now forms the comparator to arthroscopic surgery in the UK FASHIoN trial (ISRCTN64081839).

**Trial registration number:**

ISRCTN 09754699.

## Introduction

Femoroacetabular impingement (FAI) syndrome is a motion-related clinical hip disorder with a triad of symptoms, clinical signs and imaging findings.^1^ It represents a symptomatic premature contact between the proximal femur and the acetabulum.^1–4^ Typically, the morphology of the hip exhibits shapes that predispose to impingement, often described with the terms cam and pincer morphology.^2^ ^3^ ^5^ The epidemiology of cam and pincer morphology is not well defined but may be present in 30% of the general population.^5^ Not all patients with cam and/or pincer morphology develop FAI syndrome,^6^ but the treatment of those who do is controversial. Over the past 10 years, increasing numbers of patients have been treated for FAI syndrome with shape changing surgery, most frequently through hip arthroscopy.^7^ ^8^ Surgery has been shown to provide improvements in patient symptoms,^9^ although patient expectations are not always met.^10^

It has been suggested that clinicians should be cautious in the use of surgery for FAI syndrome and that non-operative approaches should be considered.^11–13^ Patients with FAI syndrome have altered hip muscle strength, range of motion (ROM) and gait biomechanics, and these offer potential targets for treatment through physiotherapy.^14–17^ While many authors recognise the likely value of non-operative or conservative care, there is very little published guidance and evidence on how this care should be delivered.^12^ ^13^

Given the uncertainty and lack of evidence about treatment for FAI syndrome, a randomised controlled trial (RCT) comparing hip arthroscopy and conservative care was proposed to guide future practice.^11^ ^18^ In 2012, the UK National Institute of Health Research (UK NIHR) Health Technology Assessment programme (HTA) commissioned us to perform a feasibility and pilot study for an RCT to compare hip arthroscopy with ‘best conservative care’ for patients with FAI syndrome (FASHIoN HTA10/41/02).^19^ At the time, there was no established ‘best conservative care’,^12^ and we are not aware of any that has been published since. To design this trial, we needed to develop a suitable conservative care protocol.

The aim of this study was to develop an agreed conservative treatment protocol for patients with FAI syndrome that was deliverable within the UK National Health Service (NHS), was safe, and that could be used in the planned RCT.

## Methods

### Study design

**Figure 1:**
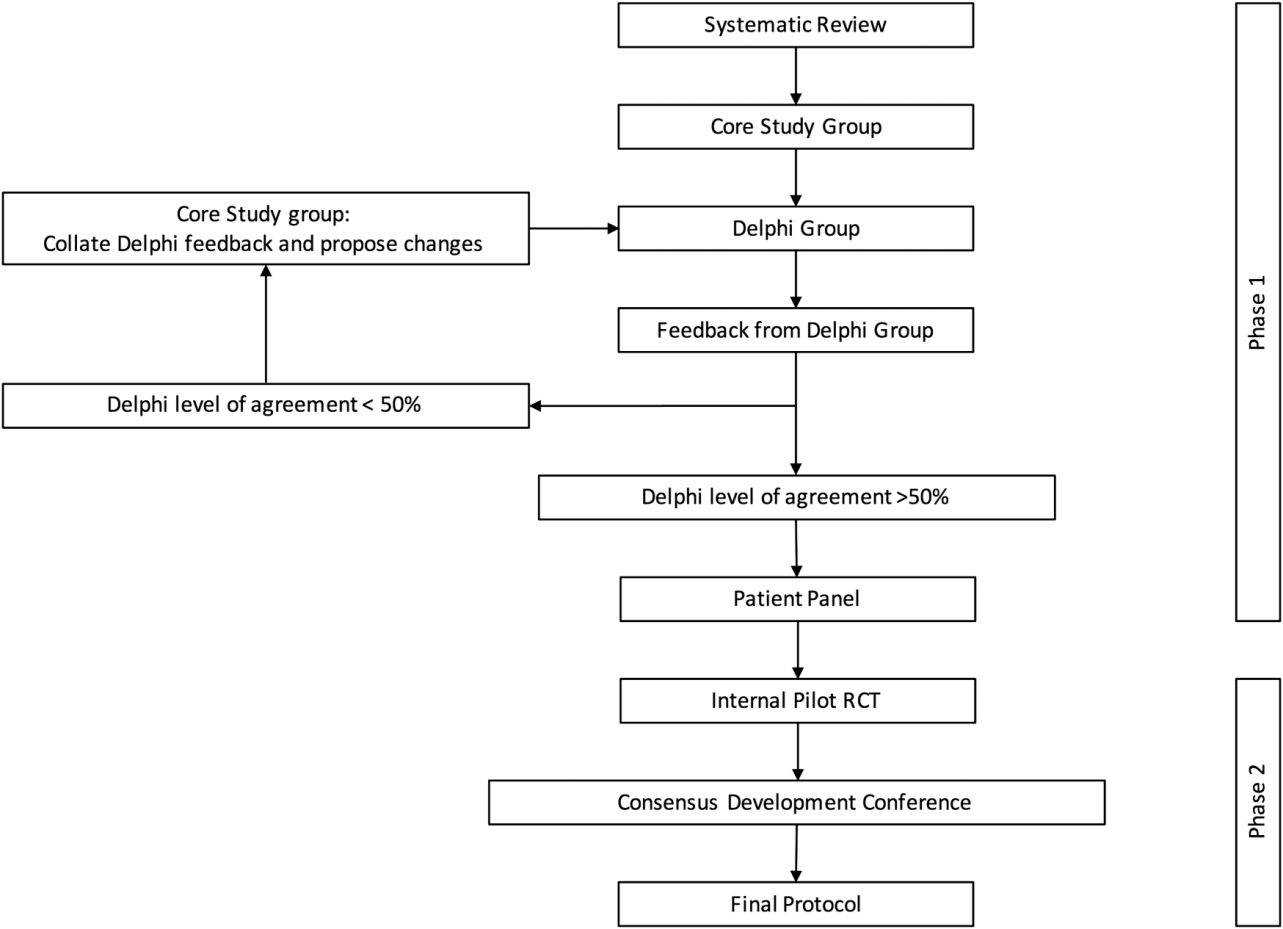
Study flow diagram. RCT, randomised controlled trial.

Research ethical approval was granted for this study (NHSREC11/WM/0389). In phase one, a non-operative treatment protocol for FAI syndrome was developed using established consensus methodology, guided by the principles described by the Medical Research Council for development of complex interventions.^20^ ^21^ In phase two, the protocol was tested in an internal pilot RCT comparing conservative care versus arthroscopic hip surgery for FAI.^22^ Figure 1 presents a flow diagram of the protocol development process. This study formed part of the FASHIoN feasibility trial whose results, including aspects of this study, have been published.^23^

#### Phase 1: consensus development

Given the lack of an agreed conservative care treatment protocol for FAI syndrome,^12^ we formed core and Delphi study groups in order to develop and agree on the treatment protocol.^20^ The core study group comprised two extended scope practitioner musculoskeletal physiotherapists with an interest in managing FAI syndrome (DR and IH), a senior academic research physiotherapist with expertise in musculoskeletal pain research (NEF) and an academic orthopaedic surgeon (PDHW). The core study group oversaw protocol development and provided the input required for consensus by nominal group technique.^20^ The use of this consensus methodology is reasonable given the lack of an established conservative care protocol.^20^ ^21^

To form the Delphi study group, we took a targeted approach to sampling, using networks of physiotherapists most likely to be involved in the management of FAI syndrome. National advertisements were placed in the orthopaedic, rheumatology, pain and manual therapy electronic networks of the Chartered Society of Physiotherapy (iCSP) and in the magazine of the Chartered Society of Physiotherapists, *Frontline*. The advertisements invited physiotherapists to help develop a consensus for a conservative care protocol for FAI syndrome. Electronic invitations were also sent to physiotherapists in the USA and Australia known to the authors through previous collaborative work on FAI syndrome. To encourage a process of ‘snowball sampling’, physiotherapists were encouraged to invite colleagues with experience and interest in managing FAI syndrome to join the consensus development process.

A systematic review was conducted to identify any previously published protocols for conservative treatment of FAI syndrome.^12^ The first protocol was drafted using evidence gathered from this systematic review and was circulated via email to the Delphi study group. They completed a questionnaire asking whether they agreed or disagreed with the proposed protocol, and where appropriate provide comments and suggestions for improvement. Additional comments and treatment strategies were grouped into themes and tabulated. An agreement level of 50% for this Delphi consensus technique was used. If no consensus was evident, the core study group refined the protocol in the light of the feedback using a nominal group technique. The refined protocol was then recirculated to the Delphi group and the cycle repeated until a consensus of at least 50% was achieved.

#### Phase 1: patient panel

As part of the FASHIoN feasibility trial, a qualitative research study among an expert patient panel was undertaken to guide many aspects of the development of the RCT.^23^ One task for the panel was to name the conservative treatment arm of the RCT. Previous qualitative research has highlighted the importance of naming treatments in order to improve uptake and adherence with treatment, in particular within the context of RCTs.^24^ Further details of this qualitative research can be found in Griffin *et al*.^23^

#### Phase 2: protocol testing and refinement

Once adequate consensus on the conservative care protocol was reached, the protocol was implemented within a multicentre internal pilot RCT comparing arthroscopic surgery versus conservative care (FASHIoN feasibility study ISRCTN09754699). Since the pilot RCT was designed to be internal to a full RCT, there was no interim analysis of outcomes; the outcomes will be reported as part of the full RCT.^22^ ^25^ The eligibility criteria for the RCT are listed in box 1.

Box 1 **Eligibility criteria for a pilot randomised controlled trial**

Inclusion criteria

- Aged ≥16; (no upper age limit)
- Symptoms of hip pain - patients may also have symptoms of clicking, catching or giving way;
- Radiographic evidence of pincer- and/ or cam-type FAI on plain radiographs and cross-sectional imaging, defined as;
  – Cam – an alpha angle >55°
  – Pincer morphology – a lateral centre edge angle of >40° or a cross over sign on the anteroposterior radiograph of the pelvis
- The treating surgeon believes that they would benefit from arthroscopic FAI surgery;
- Able to give written informed consent and participate fully in the interventions.

Exclusion criteria:

- Evidence of pre-existing osteoarthritis, defined as Tonnis grade >1, or more than 2 mm loss of superior joint space width on anteroposterior pelvic radiograph;
- Previous significant hip pathology such as Perthes’ disease, slipped upper femoral epiphysis or avascular necrosis;
- Previous hip injury such as acetabular fracture, hip dislocation or femoral neck fracture;
- Previous shape change (open or arthroscopic) in the hip being considered for treatment.

FAI, femoroacetabular impingement.

All physiotherapists delivering the conservative care protocol were asked to complete a case report form (CRF) for each patient treated, which included details about the number, nature and duration of the patient contacts, the exercises prescribed and other treatments provided. Given that this was an internal pilot trial, all patients recruited were followed up for 1 year. Any adverse events (AEs) were collated from CRFs, hospital records and follow-up questionnaires. AEs were defined as any untoward medical occurrence in the RCT.

#### Phase 2: consensus development conference

After treating patients in the internal pilot RCT, physiotherapists delivering the protocol were invited to a consensus development conference chaired by NF to share their experiences of delivering the protocol and make suggestions for further amendments prior to the full RCT.

## Results

Thirty-six physiotherapists responded and agreed to take part in the Delphi consensus process, 24 from the UK, 10 from the USA and 2 from Australia. All 36 had previously managed patients with FAI syndrome and included physiotherapists from the military, primary and secondary care, tertiary hip preservation services, extended scope practitioners, private practice and elite sport.

### Phase 1: consensus development

The first protocol proposed and circulated to physiotherapists is summarised in online supplementary file A. The level of agreement with the first protocol among the 36 physiotherapists was below the 50% threshold. Details of the degree of consensus reached and a summary of the feedback from the first round of the Delphi exercise are summarised in online supplementary file B.

10.1136/bjsports-2016-096368.supp1 Supplementary file A

10.1136/bjsports-2016-096368.supp2 Supplementary file B

Using the additional comments made by the Delphi group, and drawing on available evidence and underpinning theory, the core study group derived a second protocol. This had four core components and four optional components, which are described in table 1.

**Table 1:** Protocol components

Core 1	Patient assessment	Full history. Examination; including hip muscle stability, strength, ROM and impingement signs.	Although not formally a treatment, this component underpins the individualised treatment programme. While useful adjuncts to clinical assessment, goniometers and hand-held dynamometers were not deemed essential by the core study group. Further details of what should be included in the patient assessment are available in the PHT manual (see online supplementary file D).
Core 2	Patient education and advice	Advice about posture, gait and lifestyle behaviour modifications. Advice about activities of daily living to try to avoid FAI (reducing/avoiding deep flexion, adduction and internal rotation of hip). Advice about relative rest for acute pain.	13 additional comments from questionnaire respondents suggested that physiotherapists should provide patient specific education and advice about FAI with an indication that this should focus on lifestyle modification, advice on how to undertake different forms of exercise and how to undertake common activities such as walking, cycling, etc. Advice particularly with respect to activity modification was a feature of the published literature.^26^ ^41^ The core study group felt that education and advice would be regarded as a core component of best practice among physiotherapists managing any musculoskeletal condition. Both lifestyle and activity modification draws on relevant theory, that is, behavioural modifications that might lead to reduced functional impingement and should result in reduced symptoms.^42^
Core 3	Help with pain relief	Use of oral analgesics , including non-steroidal anti-inflammatory medication for 2–4 weeks. Engagement in and adherence to an exercise programme.	This was a feature of the first protocol, to which 44% of the physiotherapists agreed.^26^ ^41^ Analgesia is an established treatment for musculoskeletal pain.^43^ ^44^
Core 4	Exercise-based hip programme	An exercise programme that has the key features of individualisation, progression and supervision. A phased exercise programme that begins with muscle control work, and progresses to stretching and strengthening with increasing ROM and resistance. Muscle control/stability exercise (targeting pelvic and hip stabilisation, gluteal and abdominal muscles). Strengthening/resistance exercise first in available range (pain-free ROM), and targets: gluteus maximus, short external rotators, gluteus medius and abdominal muscles. Stretching exercise to improve hip external rotation and abduction in extension and flexion (but not vigorous stretching—no painful hard end stretches). Other muscles to be targeted if relevant for the patient include iliopsoas, hip flexors and rotators. Exercise progression in terms of intensity and difficulty, gradually progressing to activity or sport-specific exercise where relevant. A personalised and written exercise prescription that is progressed and revised over treatment sessions.	37 comments from questionnaire respondents endorsed hip specific and more general exercises. Of these, core or stability exercises (focusing on the activation of the hip and gluteal muscles, as well as the abdominal and paraspinal muscles targeting the restoration of control and coordination of these muscles) were the most common (n=21 additional comments). Feedback suggested that the exercise programme should be individualised to the patient (based on clinical assessment), supervised and progressed in clinic over time from core stability exercises and stretching to strengthening/resistance exercises. The exercises were to be practised at home by patients. 21 template exercises were suggested. Physiotherapists could individualise patient care by selecting a range of these to target individual movement impairments. The exercises should be progressed in terms of difficulty and intensity over time, as well as selection of different exercises to address the key findings from patient reassessment at each treatment session. The final selection of exercises is available in online supplementary file C. Exercise was a predominant feature in the published literature for managing FAI non-operatively.^26^ ^41^ ^45^ Exercise is an effective treatment for many other musculoskeletal pain problems^32^ ^33^ and exercise-based programmes can produce similar improvements in symptoms to surgery.^30^
Optional 1	Treat coexisting symptoms	Examples of this might include treating coexisting low back pain.	
Optional 2	Orthotics	Patients can be assessed for biomechanical abnormalities and have these corrected by the treating physiotherapist. Alternatively, they can be referred to other allied healthcare professionals such as podiatrists for custom-made insoles, etc.	
Optional 3	Corticosteroid hip joint injection	Potentially useful in patients who are unable to engage in the exercise-based programme due to severe pain.	
Optional 4	Manual Therapy	Hip joint mobilisations, for example, distraction, distraction with flexion, anteroposterior glides. Trigger point work.	

FAI, femoroacetabular impingement; PHT, Personalised Hip Therapy; ROM, range of motion.

The initial round of responses suggested that patients should be seen over a longer period (the first draft protocol suggested 2–3 weeks^26^) and more frequently in order to optimise patient outcomes. Within the NHS, the typical number of treatment sessions given by physiotherapists to patients with musculoskeletal pain is three or four. Evidence suggests that better outcomes are achieved from exercise-based regimes when they are supervised and the contact between the supervisor and patient is increased.^27^ ^28^ In order to allow more contact between physiotherapists and their patients, without increasing the burden of travel to clinic appointments, telephone and email contacts were also allowed to progress the exercise programme and to support patient adherence. The core study group decided that the protocol could be delivered over a 12-week period and a minimum of six treatment sessions, of which at least three should be face to face. The treatment sessions with physiotherapists were to be supplemented by patients continuing their individualised exercise programme at home. The duration of care was in keeping with established theory that suggests that physiological changes in muscle occur after a 12-week programme of exercise.^29^

The core study group agreed on the following protocol exclusions:

- Painful hard end stretches. Although only mentioned by two physiotherapists in the initial questionnaire responses, there is some evidence to suggest that painful hard end stretches and forceful manual techniques in a restricted range of movement may be harmful.^4^
- Group-based treatment was excluded to ensure that care was individualised.
- Treatment by a technical or student instructor was excluded to ensure that care was delivered by qualified physiotherapists who had received training in the protocol.

In total, 30 (83%) of the original 36 physiotherapists responded and agreed with the second protocol and provided no additional suggestions for change. One did not respond and five disagreed with the second protocol and made further suggestions for change. These points were discussed among the core study group and the following further changes were made:

- Allowing treatment to be delivered over 6 months. This was in response to concerns that the initial 12-week programme might be insufficient to correct what is likely to be a significant chronic biomechanical dysfunction. Further treatment sessions were also felt to help with patient adherence to the programme.^27^ ^28^
- The addition of taping techniques to help with postural modification and to remind patients of desired positions. Although only mentioned by one physiotherapist, it was noted that taping was a feature of the published literature.^12^ No specific protocol for taping was included; this was left to the discretion of the treating physiotherapist.
- Inclusion of an exercise diary to help patients self-monitor their home exercise programme and provide feedback to physiotherapists to guide future exercise progression.

Given the level of agreement (83%), the core study group decided to use the second protocol with the modifications discussed above for implementation in the pilot RCT.

### Phase 1: patient panel

Eighteen UK patients (8 female and 10 male) with FAI syndrome took part in the qualitative study to derive a name for the conservative care protocol, of whom 5 had been treated with conservative care and 13 with arthroscopy.^23^ They were asked to choose between four potential names which had been suggested by the core study group, with the option to suggest a different name if they wished.

Personalised Hip Therapy (HIP) appealed to and conveyed a positive message to patients. This combination emphasises that the protocol is an active intervention that differs from other conservative care regimes that patients may have previously tried.

### Phase 2: testing and refinement

Forty-two patients were recruited to the internal pilot RCT across eight NHS hospitals. The baseline patient data are displayed in table 2. Twelve experienced musculoskeletal physiotherapists delivered PHT. Two physiotherapists were Band 6, six Band 7, and two Band 8; NHS bands reflect seniority, ranging from a newly qualified physiotherapist at Band 5 to the most senior and experienced physiotherapist at Band 8. They had previously treated a mean of 30 (range 3–90) patients with FAI syndrome. Of the 42 patients recruited in the pilot RCT, 21 were allocated to PHT. On average, PHT started 38 (range 12–76) days after randomisation, reflecting physiotherapy service waiting times in the NHS. Patients attended a mean of six sessions (SD 2.3). Treatment was judged to have been delivered in line with the PHT protocol in 13 (62%) patients. Reasons for deviation from the protocol included immediate postrandomisation crossover (n=1), patient decided they no longer required treatment (n=1), no CRF received (n=2) and insufficient number of treatment sessions (n=5). Clinical outcomes are not reported from this internal pilot RCT due to the ongoing nature of the full RCT where pilot data will be included in the main analysis (FASHION ISCTRN64081839). The only AE reported was muscle soreness at 6 weeks by 13 (62%) patients.

**Table 2:** Baseline patient characteristics

Characteristics	Summary data
Age (years)*	33.4 (6.4)
Sex female: male	6: 15 (29%)
Duration of symptoms (months)*	30.9 (24.4)
UCLA score*	3.6 (2.7)
iHOT33 score*	31.4 (15.2)
SF12 PCS*	31.1 (14.8)
SF12 MCS*	46.4 (15.0)
EQ5D*	0.58 (0.23)

*mean (SD).

EQ5D, EuroQol 5 dimension questionnaire; iHOT33, internal hip outcome tool; MCS, mental component score; PCS, physical component score; SF12, short form 12; UCLA, University of California, Los Angeles activity score.

### Phase 2: consensus development conference

A consensus development conference was held at the University of Warwick in May 2013 after 21 patients randomised to PHT were treated. Eight physiotherapists from eight sites attended and provided feedback and discussion about the PHT protocol, content and delivery. Collectively, these 8 physiotherapists treated 18 patients within the pilot RCT. The physiotherapists agreed that the PHT protocol worked well, but that they would like to change the number of treatment contacts and the overall duration of the protocol. As a result, the protocol was amended to include a minimum of 6 and a maximum of 10 contacts over a total period of 6 months. In addition, 3 further exercises were recommended and were added to the original selection of 21 exercises within the exercise template (see online supplementary file C).

**Figure 2:**
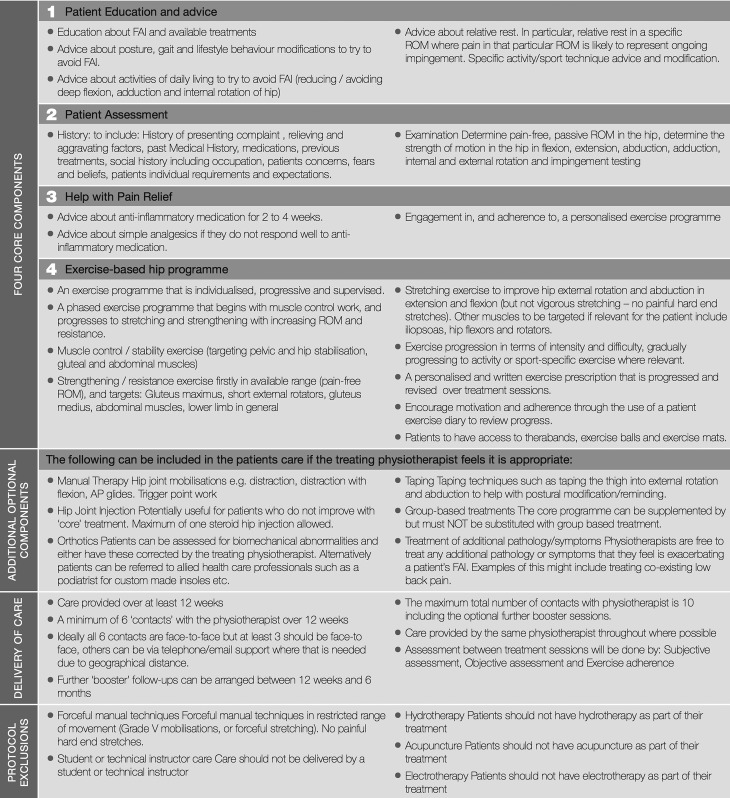
Personalised hip therapy summary. FAI, femoroacetabular impingement.

An overview of the final agreed protocol is shown in figure 2 with the exercise template provided in online supplementary file C. The full PHT manual, used as a training aid for trial physiotherapists, is available in online supplementary file D.

10.1136/bjsports-2016-096368.supp3 Supplementary file C

10.1136/bjsports-2016-096368.supp4 Supplementary file D

## Discussion

We aimed to develop and undertake initial testing of a physiotherapist-led non-operative treatment protocol for patients with FAI syndrome that could be compared with arthroscopic surgery in a large pragmatic RCT. We combined results from a systematic review, two rounds of Delphi consensus, relevant literature and the experiences of physiotherapists treating patients with FAI syndrome within the internal pilot RCT, in order to develop the agreed treatment protocol, referred to as *Personalised Hip Therapy*.^30^ ^31^ This PHT protocol provides guidance to clinicians and researchers in an area where evidence is limited.^8^ ^12^ ^18^

Exercise is an effective treatment for many types of musculoskeletal pain.^32^ ^33^ PHT has many similarities to other non-operative treatment regimens including the EULAR and OARSI guidelines on hip and knee osteoarthritis. These also recommend a comprehensive assessment, education, lifestyle modification and exercise-based programme.^34^ ^35^ However, these guidelines were unable to make specific exercise recommendations for hip OA due to a lack of evidence, although a mixed regime (quadriceps strengthening, aerobic capacity and flexibility training) was recommended in knee osteoarthritis by EULAR.^34^

As well as including aspects in common with other non-operative care protocols of hip disorders, PHT includes an exercise-based programme that aims to improve deficiencies in hip function (including muscle weaknesses and ROM) that have been highlighted in FAI syndrome.^14^ ^17^ Through an individualised exercise-based programme, physiotherapists using PHT are able to target these deficiencies. A recent editorial highlighted what might be included in a non-operative care protocol for FAI syndrome.^17^ PHT includes all of these points, including hip-specific function and lower limb strengthening, core stability and postural balance exercises.

During the pilot RCT, only 62% of PHT treatments were judged to closely follow the protocol. This raises questions about the deliverability of PHT in the ‘real world’. However, some of the reasons for deviation were teething problems to be expected in a pilot trial. These included an immediate postrandomisation crossover (n=1), no completed CRF available (n=2) and an insufficient number of treatment sessions (n=5); in some circumstances, this may reflect patient improvement, resulting in discharge prior to receiving six sessions. Compliance with home exercises can be problematic;^36^ but physiotherapists were able to use an exercise diary to monitor patient adherence to the exercise programme at home and did not report any concerns. Muscle soreness was reported in 62% of patients, 6 weeks into the PHT programme. This is to be expected as part of an exercise-based regime as the muscles adapt to the increased demand. Reassuringly, this had resolved in all cases by 3 months follow-up. This is in keeping with similar exercise-based interventions used in RCTs for other musculoskeletal conditions.^37–39^ On balance, we believe that the results from the pilot RCT show that PHT is safe and deliverable in the ‘real world’ of the NHS. The overall fidelity of PHT is similar to other conservative care protocols used in RCTs,^40^ so it is also suitable for use in the planned pragmatic RCT.

Limitations of the PHT protocol include that it was developed based on the experiences of clinicians treating patients with FAI syndrome and not by targeting the deficiencies, observed in patients with FAI syndrome, reported in the literature.

Where conservative treatment of FAI syndrome is advocated, PHT gives clinicians a protocol for content and delivery. We do not yet know how effective PHT will be—only that it is based on the published literature, represents a consensus among experienced physiotherapists on ‘best conservative care’, and that it has at least been tested in a pilot trial and found to be deliverable and safe. Following this successful pilot RCT, the effectiveness of PHT versus arthroscopic surgery is being tested in the full UK FASHIoN trial (ISCTRN64081839), where the outcome measures are hip-related quality of life (iHOT33), general health (SF12 and EQ5D) and health economics.

**Key messages**

- Femoroacetabular impingement syndrome is increasingly recognised as a source of hip pain, especially in young adults.
- A period of non-operative care is recommended, although this is poorly defined.
- A protocol for a physiotherapy-led package of education, exercise and pain relieving techniques for femoroacetabular impingement syndrome called Personalised Hip Therapy is described for use in an RCT.
- The package of care is deliverable within the UK National Health Service, is safe, and is acceptable to patients.
