# Supplementary material for: Personalised Hip Therapy: development of a non-operative protocol to treat femoroacetabular impingement syndrome in the FASHIoN randomised controlled trial
Source: Br J Sports Med. 2016 Oct;50(19):1217–23. doi: 10.1136/bjsports-2016-096368 (PMC5036255; doi:10.1136/bjsports-2016-096368)
Supplement: Supplementary file A [file bjsports-2016-096368supp_A.pdf]

Supplementary File A;

First Proposed Protocol

Table 1: First protocol

**Initial assessment and treatment**	**Stage 1**	Avoidance of excessive physical activity and use of anti-inflammatory drugs for 2 to 4 weeks
	**Stage 2**	Physiotherapy for 2 to 3 weeks in the form of stretching exercises to improve hip external rotation and abduction in extension and flexion
**Further assessment and treatment**	**Stage 3**	Assessment of the normal range of hip internal rotation and flexion after the acute pain has subsided
	**Stage 4**	Modification of activities of daily living predisposing to FAI (e.g. hip internal rotation associated with flexion and adduction)
