# Supplementary material for: Personalised Hip Therapy: development of a non-operative protocol to treat femoroacetabular impingement syndrome in the FASHIoN randomised controlled trial
Source: Br J Sports Med. 2016 Oct;50(19):1217–23. doi: 10.1136/bjsports-2016-096368 (PMC5036255; doi:10.1136/bjsports-2016-096368)
Supplement: Supplementary file B [file bjsports-2016-096368supp_B.pdf]

**Supplementary File B; Feedback from First proposed protocol**

**Level of agreement with the first protocol**

| **Agreement** | **Stage 1 and 2 of the first protocol (initial assessment and treatment)**<br>**Level of agreement n (%)** | **Stage 3 and 4 of the first protocol (further assessment and treatment)**<br>**Level of agreement n (%)** |
|---|---|---|
| **Yes** | 16 (44) | 9 (25) |
| **No** | 7 (19) | 6 (17) |
| **Unsure** | 13 (36) | 21 (58) |
| **Total** | **36** | **36** |

**Summary of additional comments made with respect to the first protocol**

| | **Additional themed comments made** | **Frequency (n)** | **Origin of comments** |
|---|---|---|---|
| Initial assessment and treatment (Stages 1 and 2) | Core stability exercise and movement control | 21 | UK x17, Australia x2, USA x2 |
| | Muscle strengthening important | 7 | UK x6 USA x1 |
| | See patients more frequently / over a longer period | 5 | UK x4 Australia x1 |
| | Stretching exercise depending on what is limited. | 4 | UK x2 USAx2 |
| | Soft tissue mobilisation to facilitate range of movement | 3 | UK x2 USA x1 |
| | Address flexion contractures | 2 | UK x2 |
| | Massage to relieve tightness in hip muscles | 2 | UK x1 Australia x1 |
| | Avoid flexion stretching exercises during initial stages | 1 | UK x1 |
| | Internal rotation stretching when pain free | 1 | USA x1 |
| | Gentle exercise to mobilise the joint in all directions | 1 | UK x1 |
| | Reduce overactive hamstrings muscles | 1 | UK x1 |
| | Work on active abduction and external rotation | 1 | UK x1 |
| | Avoid excessive hip flexion | 1 | UK x1 |
| | See patients less frequently | 1 | UK x1 |
| | Prolonged follow-up often needed | 1 | UK x1 |
| [illegible] | Advice that cycling is acceptable with activity modification | 7 | UK x 6 Australia x1 |

| | | | |
|---|---|---|---|
| | Continue strengthening | 6 | UK x6 |
| | Zigzag running no better than straight running | 4 | UK x3 Australia x1 |
| | Reassessment important | 2 | UK x2 |
| | Using orthotics may help | 2 | UK x2 |
| | Encourage hip capsule stretches | 2 | UK x2 |
| | Stretches can be harmful | 2 | UK x1 Australia x1 |
| | Identify dysfunctional movement patterns to achieve long term change | 2 | UK x1 Australia x1 |
| | More than twice monthly supervision required | 2 | UK x1 Australia x1 |
| | Advice about lifestyle modification | 2 | UK x1 USA x1 |
| | Advice about alternative forms of exercise | 2 | UK x1 Australia x1 |
| | Advise to avoid deep squatting | 1 | UK x1 |
| | Advice on return to sport specific training | 1 | Australia x1 |
| | Strengthening of internal and external rotators of hip | 1 | UK x1 |
| | Activity restriction on an individual basis | 1 | UK x1 |
| | Modification of running on an individual basis | 1 | UK x1 |
| | Ensure activities can be undertaken with minimal adduction/Internal rotation | 1 | UK x1 |
