# Supplementary material for: Personalised Hip Therapy: development of a non-operative protocol to treat femoroacetabular impingement syndrome in the FASHIoN randomised controlled trial
Source: Br J Sports Med. 2016 Oct;50(19):1217–23. doi: 10.1136/bjsports-2016-096368 (PMC5036255; doi:10.1136/bjsports-2016-096368)
Supplement: Supplementary file C [file bjsports-2016-096368supp_C.pdf]

Personalised Hip Therapy
Core Exercises

1. Gym Ball Exercise

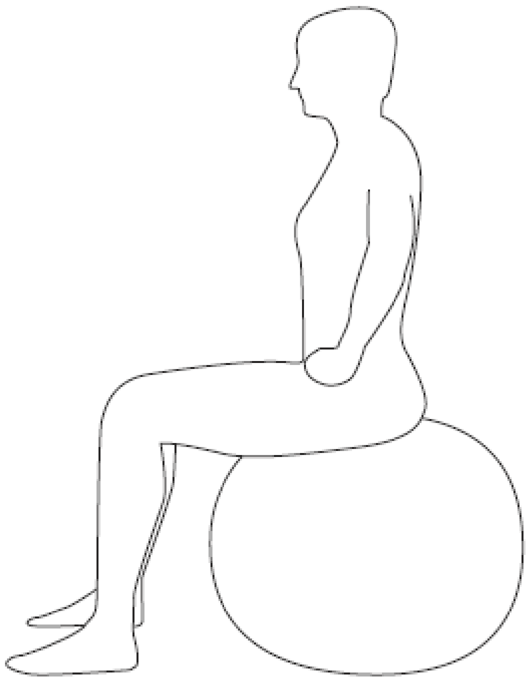

Description

- Practise sitting on the ball and gently moving your pelvis forwards and backwards, side to side and in circles.

Prescription

To be customised for the patient and progressed. E.g. duration, frequency, repeats, addition of theraband etc.

Duration ___________________________________

Frequency ___________________________________

Additional information ___________________________________

Extra option 1

- Lift one foot from the floor whilst maintaining your balance and keeping good symmetrical posture on the ball.
- Relax and repeat on alternate legs.

Your Physiotherapist is: ______________________
Contact Number is: ______________________

2. Abdominal Exercise

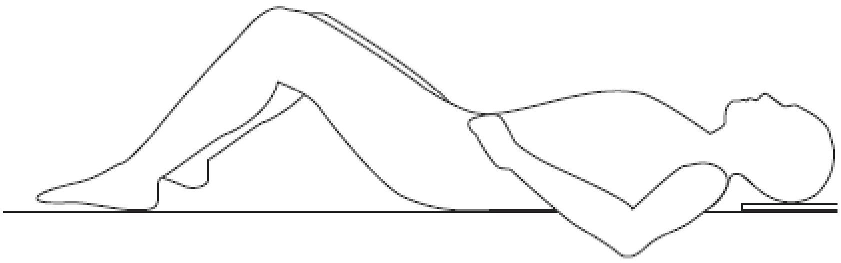

Description

- On your back, draw the belly button down towards the spine
- Keep pelvis still and keep breathing!

Prescription

To be customised for the patient and progressed. E.g. duration, frequency, repeats, addition of theraband etc.

Duration ____________________________________

Frequency ____________________________________

Additional information ____________________________________

Your Physiotherapist is: ______________________
Contact Number is: ______________________

3. Stability Exercise q

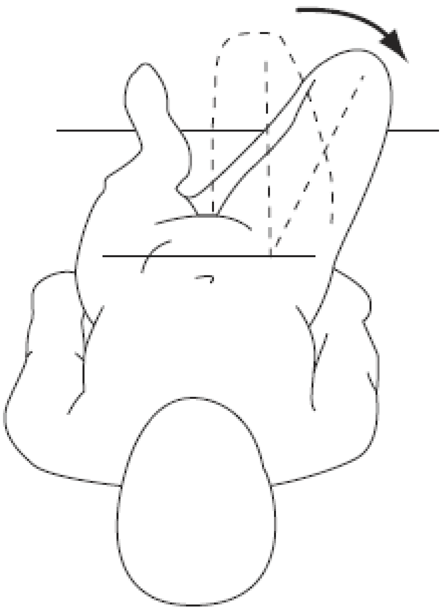

Description

- Lie on back with legs bent, feet together and flat on the bed.
- Keep the lower back in neutral position throughout the exercise.
- Tighten the lower stomach muscles as described in abdominal exercise.
- Sustain throughout the exercise.
- Keeping the heel on the floor slowly let one hip roll out to the side.
- Only roll the leg as far as trunk control allows.
- Slowly return to the start position with control.
- Make sure that you keep the muscle at the front of your hip relaxed.
- Repeat on the opposite side
- Repeat ____ times on each leg.

Prescription

To be customised for the patient and progressed. E.g. duration, frequency, repeats, addition of theraband etc.

Duration ___________________________________

Frequency ___________________________________

Additional information ___________________________________

Your Physiotherapist is: ______________________
Contact Number is: ______________________

4. Stability Exercise r

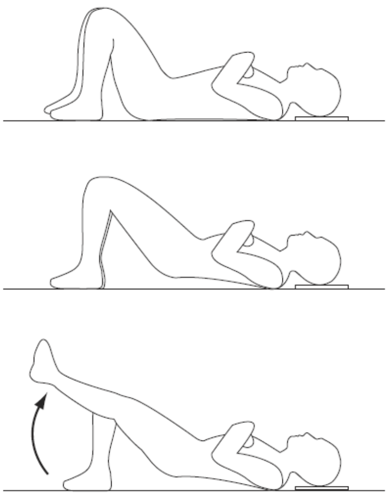

Description

- Lie on the back with knees bent and feet slightly apart.
- Tighten the lower stomach muscles as described in abdominal exercise.
- Keeping the back flat slowly lift the pelvis until your knees, hips and shoulders are level.
- Hold this position for _____seconds, relax slowly.
- Repeat up to ____ times.
- You should feel your bottom and stomach muscles doing the work and not the hamstring muscles at the back of the thigh.

Prescription

To be customised for the patient and progressed. E.g. duration, frequency, repeats, addition of theraband etc.

Duration ___________________________________

Frequency ___________________________________

Additional information ___________________________________

Extra Option 1

- Same as above but when you have your bottom lifted try lifting one heel at a time from the bed whilst keeping your pelvis still.
- Hold this position for ___ seconds, relax slowly.
- Repeat up to ____ times.

Extra Option 2

- Same as above but then shift the weight onto one foot and slowly straighten the other knee without side shifting or twisting the pelvis excessively.
- The knees should stay slightly apart.
- Slowly return to the start position with control.
- Hold this position for ___ seconds, relax slowly.
- Repeat up to ___ times.

5. Stability Exercise t

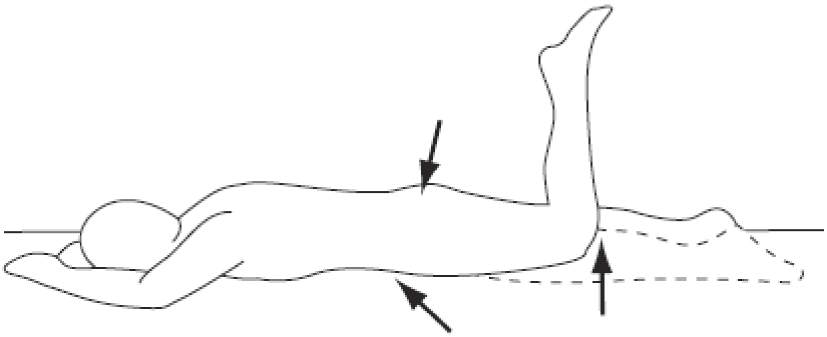

Description

- Lie face down with hips and back relaxed and one knee bent.
- Tighten the lower stomach muscle and gently squeeze both buttocks to flatten the lower back.
- Hold this contraction and lift the bent leg 5cm.
- Do not let the back arch or pelvis twist.
- Hold for ____ seconds.
- Repeat ____ times on each leg.

Prescription

To be customised for the patient and progressed. E.g. duration, frequency, repeats, addition of theraband etc.

Duration ____

Frequency ____

Additional information ____

Your Physiotherapist is: ________________________
Contact Number is: ________________________

6. Stability Exercise o

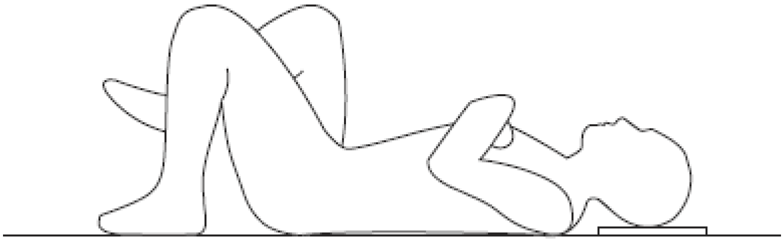

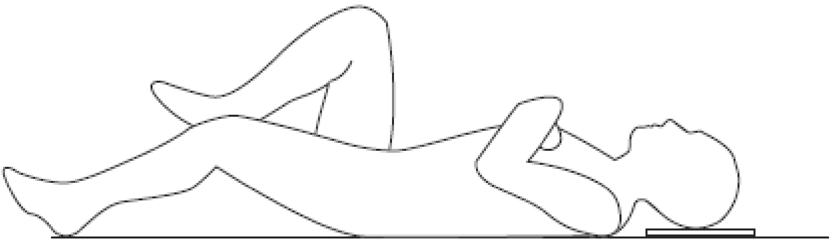

Description

- Lie on back with legs bent, feet together and flat on the bed.
- Keep the lower back in a neutral position throughout the exercise.
- Tighten the lower stomach muscles as described in abdominal exercise and sustain throughout the exercise.
- Keeping the heel on the floor, slowly slide the leg out
- Only slide the leg as far as trunk control allows.
- Slowly return to the start position with control.
- Repeat on the opposite side.
- Repeat ___ times on each leg.

Prescription

To be customised for the patient and progressed. E.g. duration, frequency, repeats, addition of theraband etc.

Duration ___________________________________

Frequency ___________________________________

Additional information ___________________________________

Your Physiotherapist is: ______________________
Contact Number is: ______________________

7. Stability Exercise p / Clam

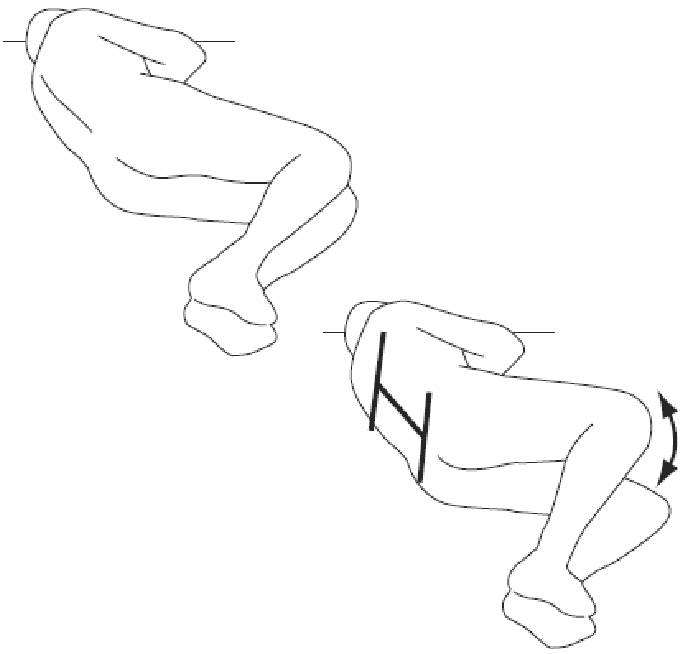

Description

- Lie on the side with pelvis square, the hips flexed to approximately 45 degrees and the knees bent.
- Tighten the lower stomach muscles as in exercise 1 to maintain the back to a neutral position throughout the exercise.
- Leaving the heels together slowly lift the top knee by turning the hip out without letting the back or pelvis twist.
- Only turn out as far as stable back and pelvis allow.
- Hold this position with minimal effort.
- Hold for ___ seconds
- Repeat ___ times.

Prescription

To be customised for the patient and progressed. E.g. duration, frequency, repeats, addition of theraband etc.

Duration ___________________________________

Frequency ___________________________________

Additional information ___________________________________

Your Physiotherapist is: ______________________
Contact Number is: ______________________

8. Stability Exercise s

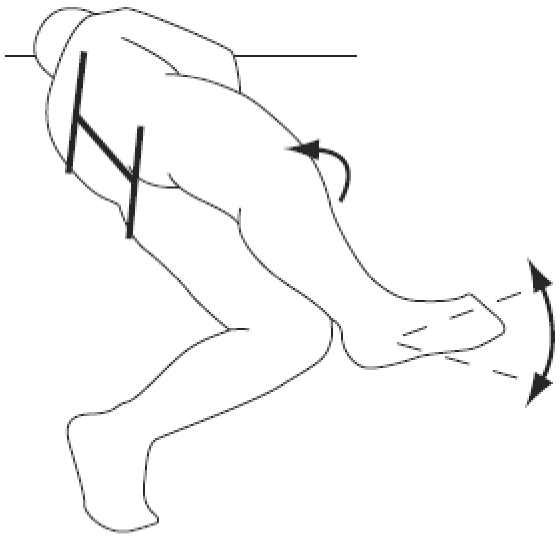

Description

- Lie on your side with the pelvis square.
- The bottom leg is comfortably bent while the top leg is held straight.
- Tighten the lower stomach to keep the back in a neutral position throughout the exercise.
- Keep the knee facing forward / out.
- Lift the leg up and back slightly.
- Hold this position for a few seconds then slowly turn the leg back in.
- Only turn out as far as a stable back and pelvis allow.
- Hold for ___ seconds.
- Repeat ___ times.

Prescription

To be customised for the patient and progressed. E.g. duration, frequency, repeats, addition of theraband etc.

Duration ____________________________________

Frequency ____________________________________

Additional information ____________________________________

Your Physiotherapist is: ______________________
Contact Number is: ______________________

9. Stability Exercise g

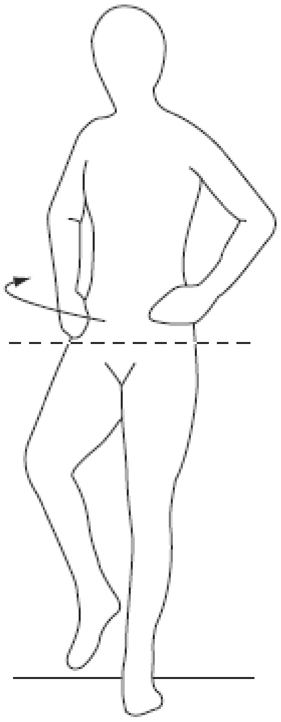

Description

- Stand tall with feet together and weight over the mid foot (do not let the pelvis sway forward).
- Gently squeeze both buttocks then slowly lift one foot behind you (5cms).
- Keep the shoulders and pelvis level
- Slowly turn body away from the weight bearing leg.
- Do not allow any forward or sideways tilt of the pelvis.
- Make sure the buttock on the weight bearing leg remains tight.
- Hold for ___ seconds.
- Repeat___ times

Prescription

To be customised for the patient and progressed. E.g. duration, frequency, repeats, addition of theraband etc.

Duration ___________________________________

Frequency ___________________________________

Additional information ___________________________________

Your Physiotherapist is: ______________________
Contact Number is: ______________________

10. Stability Exercise h

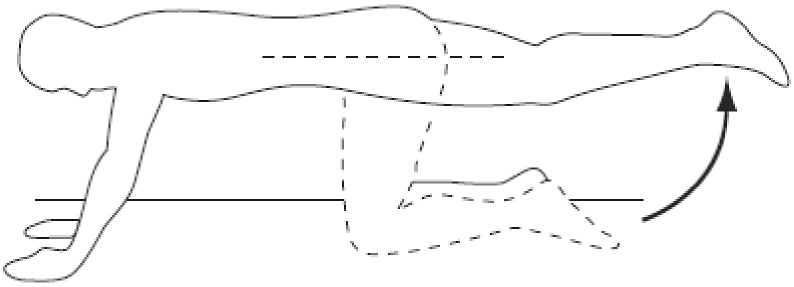

Description

- On hands and knees with the knees under the hips and the back relaxed in a neutral position.
- Keep the back controlled, slowly straighten the hip and knee to lift the leg out behind you.
- Contract the buttocks slightly during the leg lift.
- Do not let the back arch or twist.
- Only lift as far as the stable back allows.
- Hold for ___ seconds then lower slowly.
- Repeat ___ times.

Prescription

To be customised for the patient and progressed. E.g. duration, frequency, repeats, addition of theraband etc.

Duration ___________________________________

Frequency ___________________________________

Additional information ___________________________________

Your Physiotherapist is: ______________________
Contact Number is: ______________________

11. Stability Exercise L / Forward Lunge

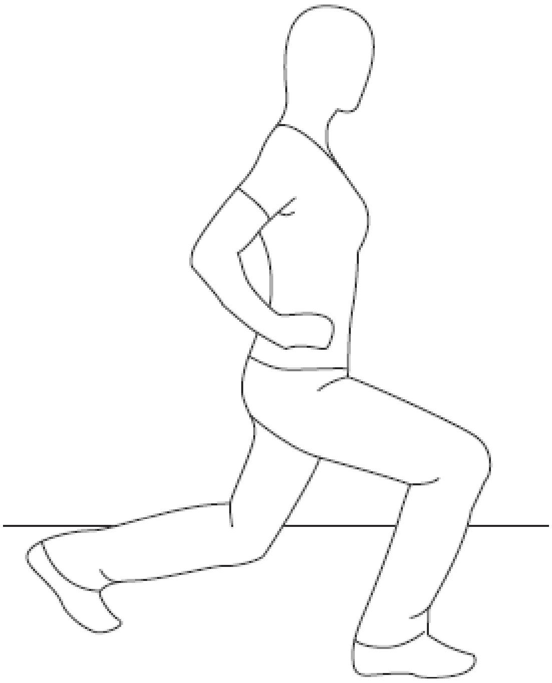

Description

- Stand straight.
- Take a step forward and bend your knees.
- Return to the starting position.
- Repeat ___ times.

Prescription

To be customised for the patient and progressed. E.g. duration, frequency, repeats, addition of theraband etc.

Duration ___________________________________

Frequency ___________________________________

Additional information ___________________________________

Your Physiotherapist is: ______________________
Contact Number is: ______________________

12. Stability Exercise z

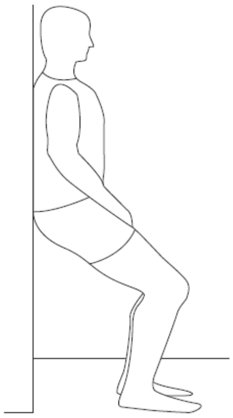

Description

- In standing, back against a wall.
- Slide down wall about 6-8 inches.
- Keep back against the wall.
- Repeat ___ times.

Prescription

To be customised for the patient and progressed. E.g. duration, frequency, repeats, addition of theraband etc.

Duration ___________________________________

Frequency ___________________________________

Additional information ___________________________________

Extra Option 1

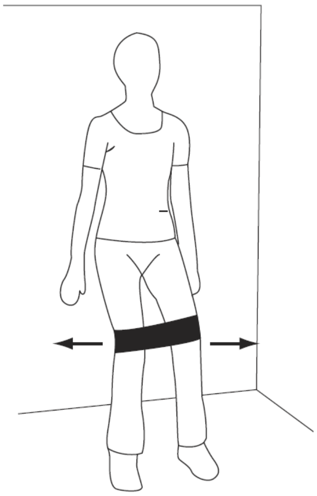

- Place a theraband around your knees.
- Try to separate your knees against the resistance of the theraband. At the same time slide down wall about 6-8 inches and then relax the theraband.
- Repeat ___ times.

Your Physiotherapist is: ______________________

Contact Number is: ______________________

13. VMO Exercise

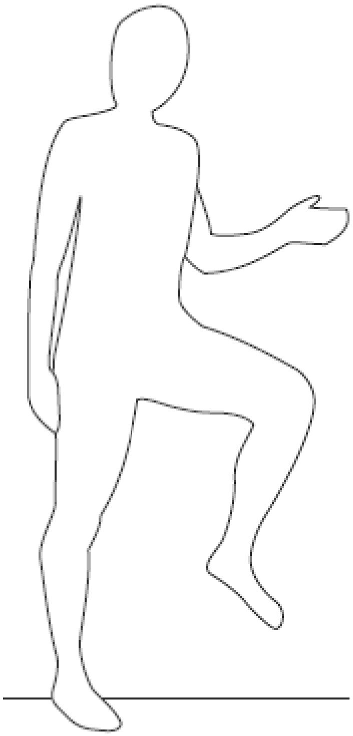

Description

- Lean sideways against the wall, standing on your operated leg.
- Bend the non-effected leg up against the wall in front of you.
- Use the non-effected leg to push against the wall while keeping your balance with the muscles of the operated leg.
- Hold for ___ seconds.
- Repeat ___ times.

Prescription

To be customised for the patient and progressed. E.g. duration, frequency, repeats, addition of theraband etc.

Duration ___________________________________

Frequency ___________________________________

Additional information ___________________________________

Extra Option 1

- Same as above
- Add in an exercise ball between the non-effected leg (knee) and the wall, while keeping your balance with the muscles of the operated leg.

Your Physiotherapist is: ______________________
Contact Number is: ______________________

14. Bottom Lift

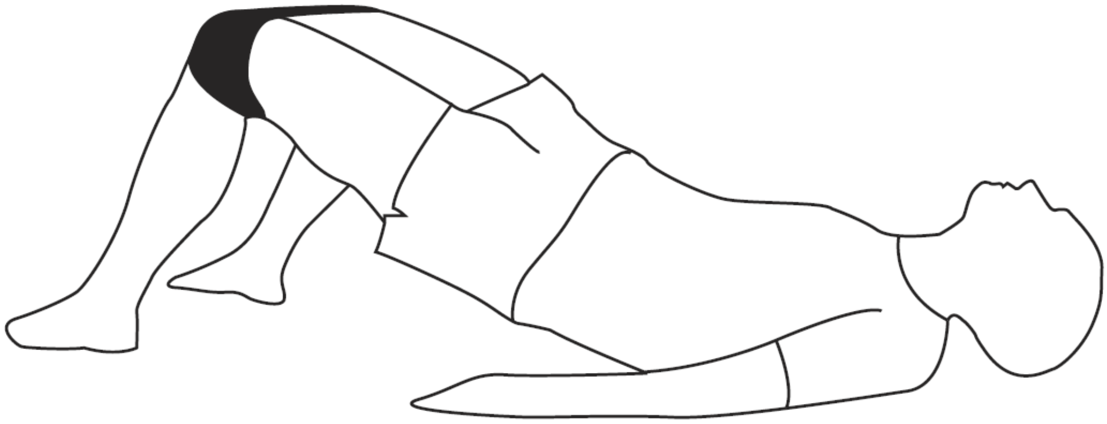

Description

- Lie on your back, hip and knees bent, theraband around your knees
- Lift hips whilst keeping knees apart
- Lower keeping knees apart.
- Repeat ___ times.

Prescription

To be customised for the patient and progressed. E.g. duration, frequency, repeats, addition of theraband etc.

Duration ____________________________________

Frequency ____________________________________

Additional information ____________________________________

Your Physiotherapist is: ______________________
Contact Number is: ______________________

15. Inverted Hamstring Strength Exercise

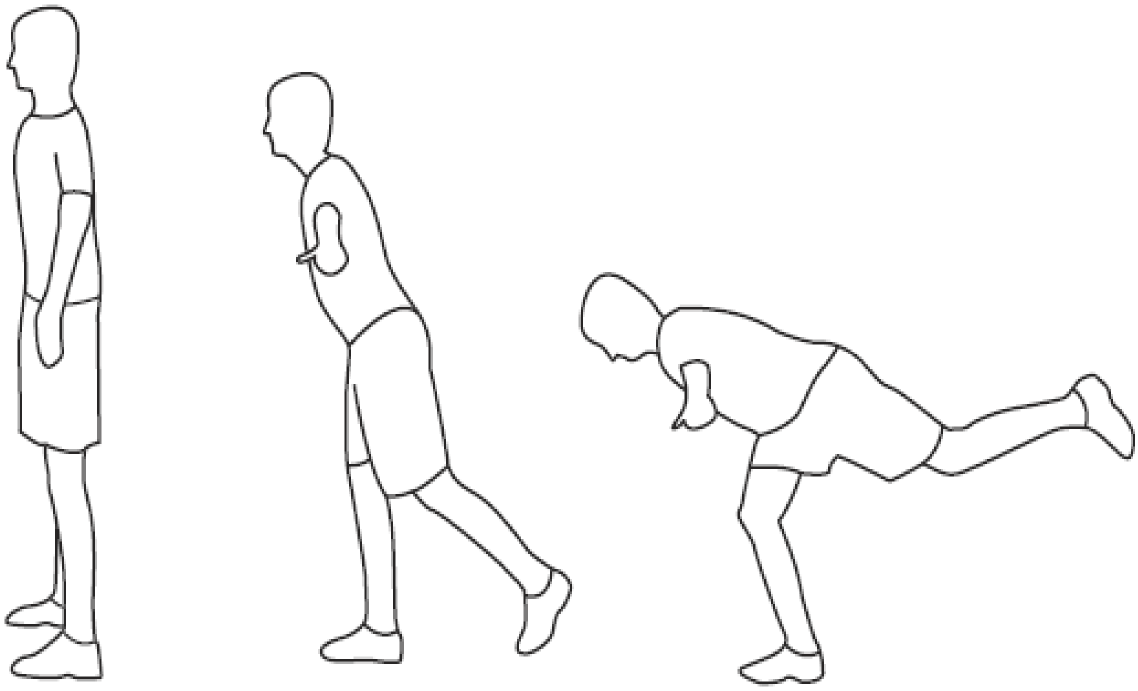

Description

- Straight arms.
- Maintain core.
- Fully extend hip and knee.
- Hold for ___ seconds.
- Aim to do ___ repeats.

Prescription

To be customised for the patient and progressed. E.g. duration, frequency, repeats, addition of theraband etc.

Duration ___________________________________

Frequency ___________________________________

Additional information ___________________________________

Your Physiotherapist is: ______________________
Contact Number is: ______________________

16. 3 Way Lunge Strength Exercise

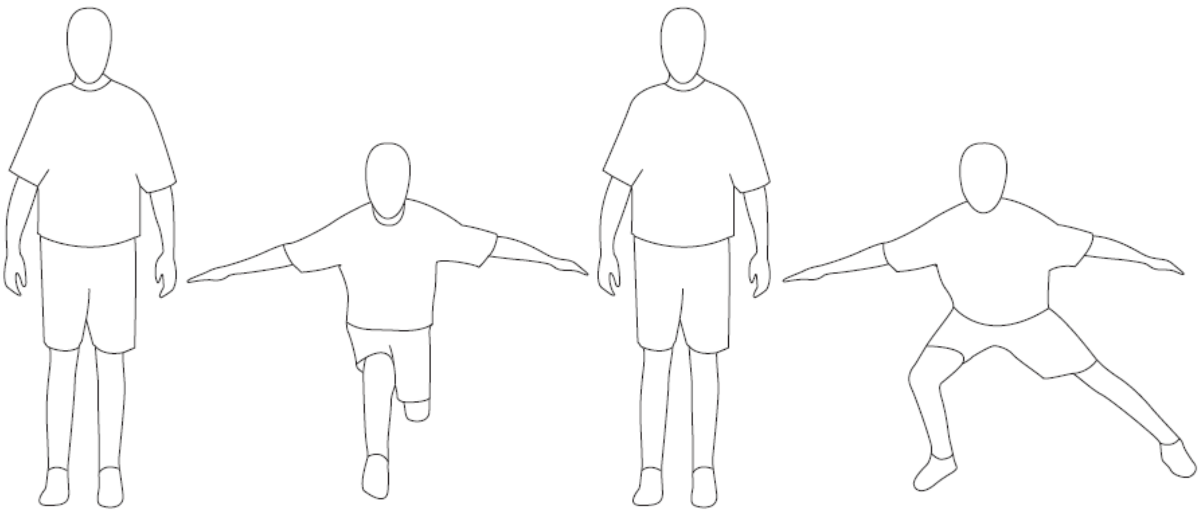

Description

- From starting position, lunge forward, then straight backward then to side
- ___ times through, so in effect ___ toe touches on each leg

Prescription

To be customised for the patient and progressed. E.g. duration, frequency, repeats, addition of theraband etc.

Duration ________________________________

Frequency ________________________________

Additional information ________________________________

Your Physiotherapist is: ____________________
Contact Number is: ____________________

17. Crab Walk Strength Exercise

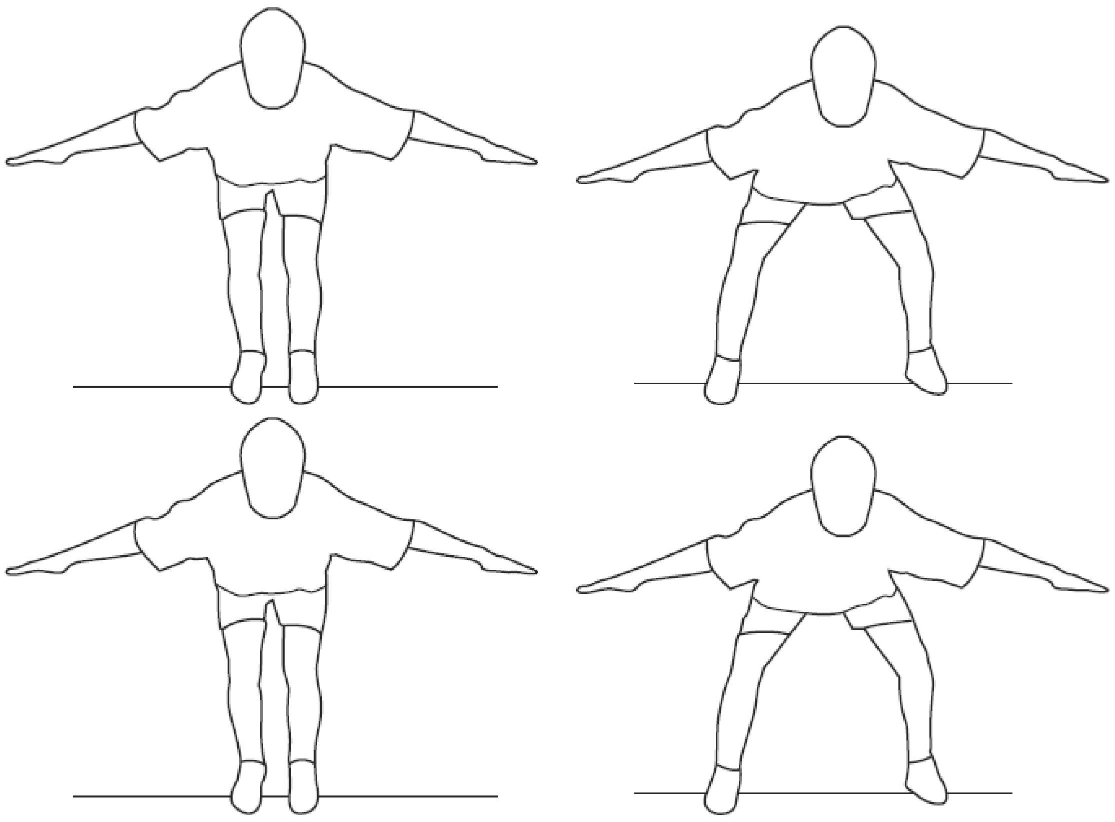

Description

- 15m long line
- Stay low in squat position
- Side step both directions

Prescription

To be customised for the patient and progressed. E.g. duration, frequency, repeats, addition of theraband etc.

Duration ___________________________________

Frequency ___________________________________

Additional information ___________________________________

Your Physiotherapist is: ______________________
Contact Number is: ______________________

18. Gluteal Dip Strength Exercise

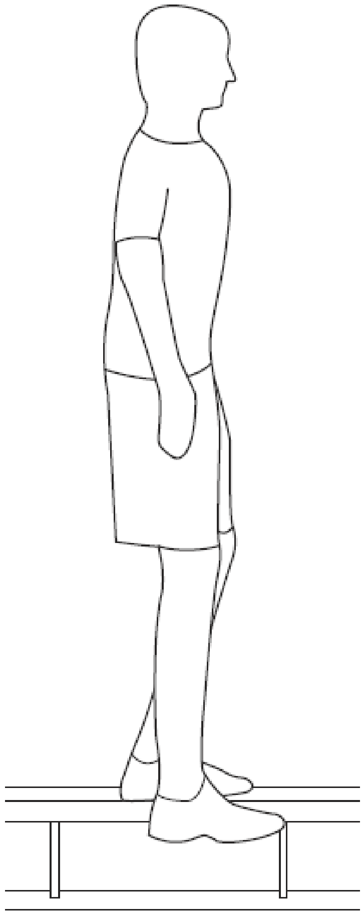

Description

- Stand on a raised stable surface, with a straight back.
- Stand on one leg, with the free leg straight and off the edge of the surface.
- Allow the free leg to dip down below the surface 5-10cm.
- Do the same on the other leg.
- Should feel “burn” in standing leg around the buttock area.

Prescription

To be customised for the patient and progressed. E.g. duration, frequency, repeats, addition of theraband etc.

Duration ______________________________

Frequency ______________________________

Additional information ______________________________

Your Physiotherapist is: ____________________
Contact Number is: ____________________

19. Side Plank

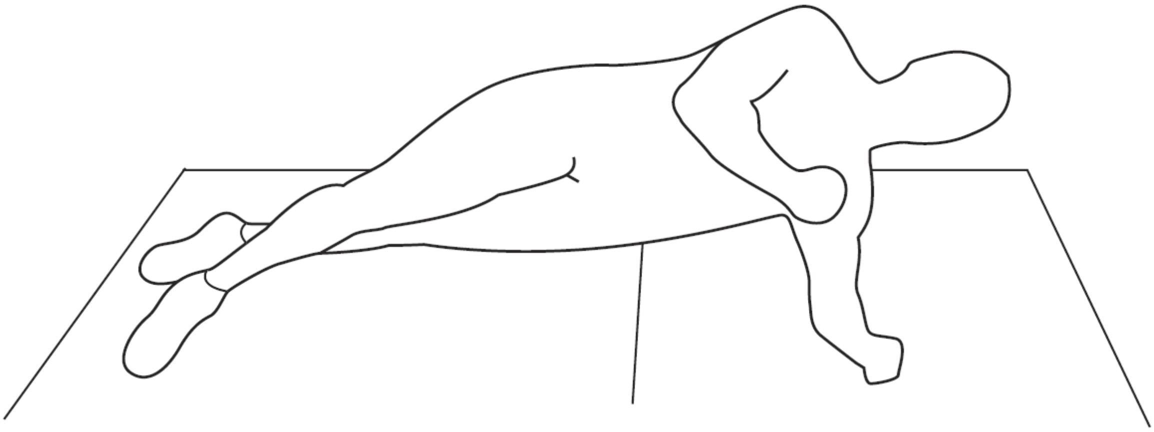

Description

- Lying on your side, elbow of bottom arm on the floor, and top leg in front of bottom leg.
- Raise your hips up so that your spine is like a board.
- Do not let your legs sag down or backwards.
- Hold for ___ seconds.

Prescription

To be customised for the patient and progressed. E.g. duration, frequency, repeats, addition of theraband etc.

Duration ___________________________________

Frequency ___________________________________

Additional information ___________________________________

Your Physiotherapist is: ______________________
Contact Number is: ______________________

20. Kneeling / Hip Flexor Stretching

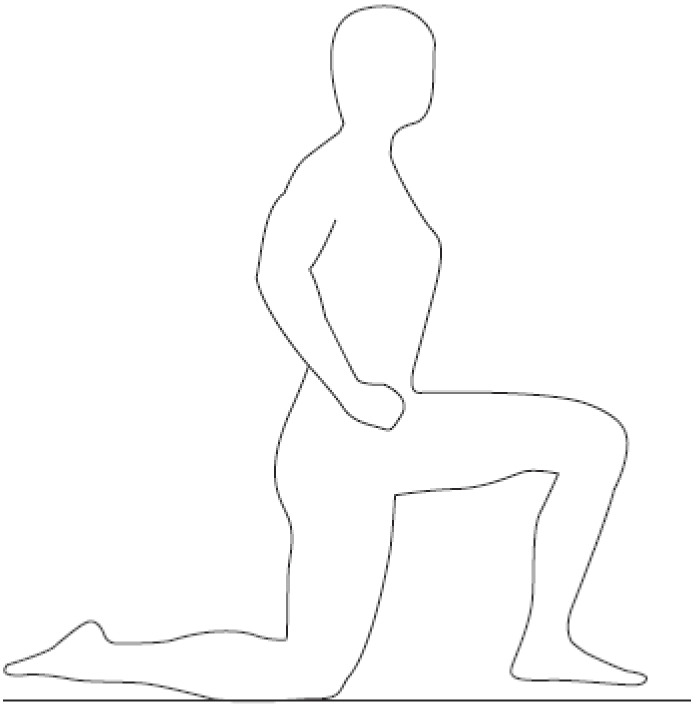

Description

- Kneel on the floor with your trunk upright.
- Gently lean back at the waist until you feel a pull on the front of your thighs.
- Hold for ___ seconds.
- Repeat ___ times.

Prescription

To be customised for the patient and progressed. E.g. duration, frequency, repeats, addition of theraband etc.

Duration ____________________________________

Frequency ____________________________________

Additional information ____________________________________

Your Physiotherapist is: ______________________
Contact Number is: ______________________

21. External Rotation Stretches

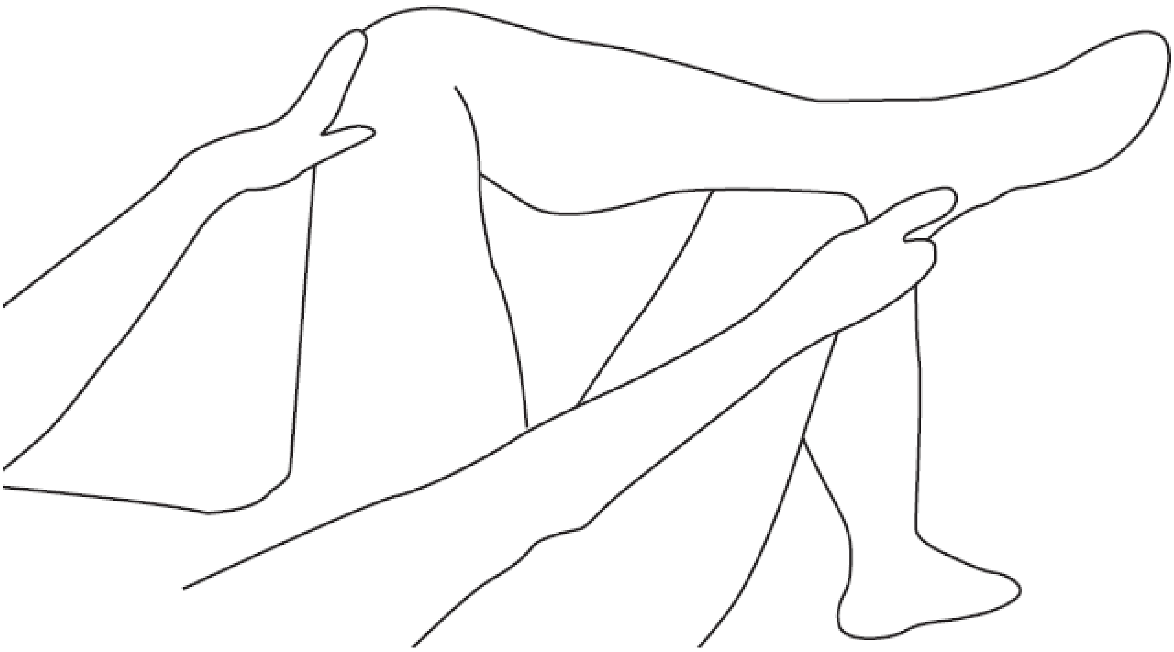

Description

- On your back with hips bent, place effected legs ankle onto opposite knee (as shown).
- Slowly bend non-effected hip and feel pull in buttock.
- Hold for ___ seconds.
- Repeat __ times.

Prescription

To be customised for the patient and progressed. E.g. duration, frequency, repeats, addition of theraband etc.

Duration ___________________________________

Frequency ___________________________________

Additional information ___________________________________

Your Physiotherapist is: ______________________
Contact Number is: ______________________

22. Pelvic tilt exercises

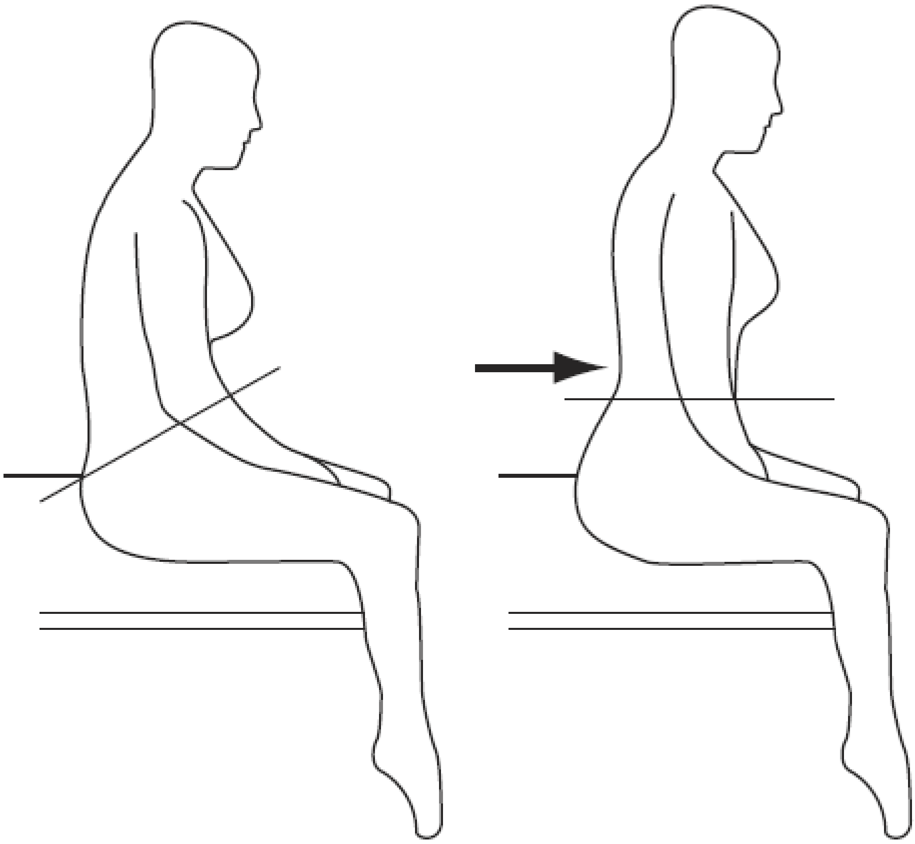

Description

- Sit comfortably with knees bent over the edge of a chair or table
- Allow abdominal, pelvic and lower back muscles to relax
- Then tighten the lower back and pelvis muscles in order to exaggerate the lumbar curvature of the lower back and bring the pelvis into a more upright position (see second diagram).
- Hold for ___ seconds
- Then relax back

Prescription

To be customised for the patient and progressed. E.g. duration, frequency, repeats, addition of theraband etc.

Duration ___________________________________

Frequency ___________________________________

Additional information ___________________________________

23. Anterior capsule hip joint mobilisations

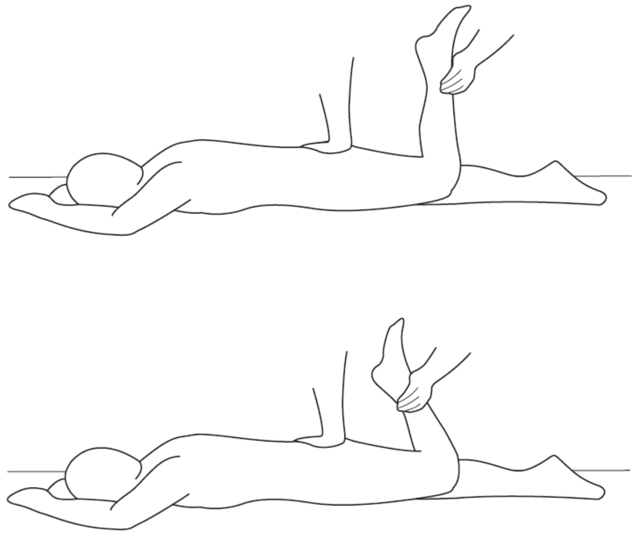

Description

- Patient lying prone
- You may place a rolled up towel under thigh to bring hip into slight extension
- Place hand over the posterior aspect of the greater trochanter
- You may need to flex the knee to wind up rectus femoris and tighten anterior capsule (second diagram).
- Use your hand to push and mobilise the femoral head in a posterior anterior direction.
- N.B You can add in external rotation to wind the anteromedial capsule up before applying the posterior anterior mobilisation.

Prescription

Not applicable

Your Physiotherapist is: ______________________
Contact Number is: ______________________

24. Hip joint glides

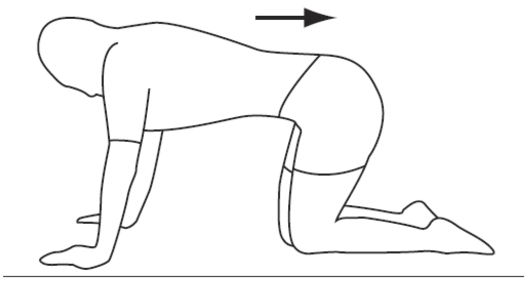

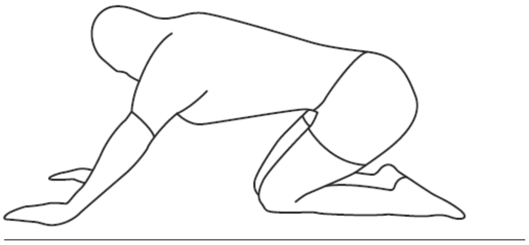

Description

- On all fours with arms and thighs vertical
- Gently rock backwards so that arms are in front and the knees bend
- Rock backwards as far as is comfortable and hold for ___ seconds
- Return to starting position
- Repeat ___ times

Prescription

To be customised for the patient and progressed. E.g. duration, frequency, repeats, addition of theraband etc.

Duration ___________________________________

Frequency ___________________________________

Additional information ___________________________________
