# Supplementary material for: Personalised Hip Therapy: development of a non-operative protocol to treat femoroacetabular impingement syndrome in the FASHIoN randomised controlled trial
Source: Br J Sports Med. 2016 Oct;50(19):1217–23. doi: 10.1136/bjsports-2016-096368 (PMC5036255; doi:10.1136/bjsports-2016-096368)
Supplement: Supplementary file D [file bjsports-2016-096368supp_D.pdf]

UK Full Randomised Controlled Trial of Arthroscopic Surgery for Hip Impingement versus best CoNventional Care

REC 14/WM/0124 I HTA 13/103/02 I ISRCTN64081839

FASHION
STUDYING HIP IMPINGEMENT

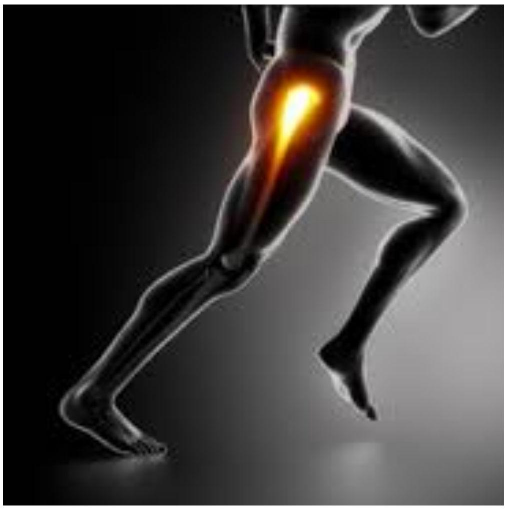

PERSONALISED HIP THERAPY

The Manual

Warwick Medical School
CLINICAL TRIALS UNIT

Keele University

NHS
National Institute for Health Research

Contents

1. Contact Details 4
2. Background 5
3. Feasibility and pilot studies 6
4. Scientific Plausibility of Personalised Hip Therapy (PHT) 6
5. Effectiveness of other physiotherapy regimes 7
6. Define a protocol for best conventional care (comparator) 7
7. Personalised Hip Therapy (PHT) 8
FOUR Core Components 9
7.1 Patient Education and advice 9
7.2 Patient Assessment 9
7.3 Help with Pain Relief 10
7.4 Exercise-based hip programme 10
8. Delivery of care 11
9. Additional optional components 12
10. Protocol exclusions 12
11. Comments and Suggestions 12
10. References 13

1. Contact Details

NOT DISPLAYED

2. Background

Until recently, there was little understanding of the causes of hip pain in young adults. A few of these patients had established osteoarthritis, inflammatory arthritis, avascular necrosis, fractures or childhood hip disease, but the majority had no specific diagnosis. In the last few years there has been increasing recognition of the syndrome of FAI, which seems to account for a large proportion of the previously undiagnosed cases of hip pain in young adults.[1,2] Subtle deformities in the shape of the hip (ball and socket joint) combine to cause impingement between femoral head (ball) or neck and the anterior rim of the acetabulum (socket), most often in flexion and internal rotation.[1,3] Excess contact forces lead to damage to the acetabular labrum (fibrocartilage rim of the socket) and the adjacent acetabular cartilage surface.[1] FAI seems to be associated with progressive articular degeneration of the acetabulum and may account for a significant proportion of so called idiopathic osteoarthritis, although this remains unproven.[3] The shape abnormalities of the hip joint are typically divided into three categories:[3]

- Cam-type, in which the femoral head is oval rather than round, or there is prominent bone on the femoral neck;
- Pincer-type, in which the rim of the acetabulum is too prominent, in one or more areas of its circumference;
- Mixed-type hip impingement, which is a combination of cam and pincer types.

Surgery can be performed to improve bone shapes in order to prevent impingement between the femoral neck and rim of the acetabulum. In the case of cam-type FAI this usually involves removal of bone at the femoral head-neck junction. In the case of pincer-type FAI, it may involve removal of bone at the rim of the acetabulum. At the same time as bony shape improvement, any soft tissue damage to the cartilage or labrum as a result of the FAI is debrided, repaired or reconstructed. Surgery can be undertaken using either keyhole (arthroscopic surgery) or more traditional open surgery to access the hip joint and correct the hip shape abnormalities associated with FAI.

Surgery for FAI has evolved more quickly than our understanding of the epidemiology or natural history of the condition[4-8], yet it is becoming an established treatment within the NHS. The risks of complications from open surgery are greater than those for arthroscopic surgery[9] and current evidence suggests that the outcomes of arthroscopic treatment for the symptoms of FAI are comparable to open surgery.[10] Consequently, hip arthroscopy for FAI is a rapidly growing new cost pressure for the NHS. Three systematic reviews have shown that no RCTs have been conducted to measure the clinical or cost effectiveness of either surgery or non-operative care for FAI[8,11-13], and we have recently confirmed this in a Cochrane systematic review (not yet published). In particular there is no RCT of hip arthroscopy compared with conventional care in patients with FAI.

Multi-centre randomised controlled trials (RCTs) are acknowledged to be the best design for evaluating the effectiveness of health care interventions as they provide robust evidence.[14,15] However, there are often major challenges in performing RCTs of surgical technologies[25], and there have been concerns that an RCT of hip arthroscopy in FAI might not be feasible.

3. Feasibility and pilot studies

A feasibility and pilot study commissioned by HTA (HTA 10/41) has been completed. It comprised: (i) a pre-pilot phase including patient and clinician surveys and interviews, and a systematic review of non-operative care; (ii) a workload survey of hip arthroscopy for FAI; (iii) development of best conventional care and arthroscopic surgery protocols; (iv) a pilot RCT to measure recruitment rate; and (v) an integrated programme of qualitative research (IQR) to understand and optimise recruitment.

4. Scientific Plausibility of Personalised Hip Therapy (PHT)

The symptoms of FAI are believed to be the result of impingement between the femoral head and the acetabulum (the hip ball and socket joint) due to abnormalities of shape and pelvic orientation. These abnormalities are thought to predispose to early and repetitive contact between the femoral head and the acetabular labral and articular surfaces during movement of the hip joint. This contact may cause damage to the soft tissues around the hip including the labrum (cushion around the hip, which then causes pain.

The personalised hip programme (PHT) has two goals:

- Control and reduce symptoms
- Prevent recurrence of symptoms

The programme will achieve this by teaching patients new techniques and ways of moving during everyday tasks and leisure activities to reduce and avoid hip impingement. PHT will focus on improving the stability and fine control of movement around the hip, as well as improving the strength and flexibility of the joints and muscles close to the hip. Through this PHT patients will be equipped with the right knowledge and skills to modify and maintain ways of moving to reduce and avoid impingement. These improved movement patterns are consciously learnt to begin with but become routine with practice over time.

The programme will provide patients with a high level of understanding of their FAI. Following a detailed assessment the physiotherapist will prescribe a personalised rehabilitation programme that is individualised to each patient's clinical presentation, activity levels and expectations. Over a series of between 6 to 10 treatment contacts, the programme will be progressed guided by the individual requirements and progress of each patient.

Any damaged soft tissues including the labrum that are so acutely painful that engagement in exercise is impossible will be subjected to a period of relative rest with the PHT. Soft tissues including the labrum have the ability to heal naturally, which can take up to several weeks or months. After this period provided that the aggravating impingement has been reduced by improved hip and local joint control methods further painful impingement will be reduced. This is thought to prevent further progress of the FAI.

Several studies have shown the lack of clear association between imaging findings and clinical symptoms of pain and functional limitations. For example studies have found MRI abnormalities in people without any pain and some people with severe knee pain do not have observable x-ray abnormalities. Not much is yet known about the influence of the soft tissues

(particularly muscles) in the symptoms of FAI[16,] but some evidence concluded that there is a significant soft tissue component in FAI and that it may involve other joints including the lumbo-sacral joint.[17] Postural abnormalities and muscular imbalances are clear targets for treatment in order to prevent recurrence of FAI symptoms. For example shortening of hip flexors and erector spinae is accompanied by weakness in gluteal and abdominal muscles. These lead to increased anterior tilt in the pelvis and this may contribute to abnormal positioning of the acetabulum and abnormal load transmission across the hip. Other presentations may include long and weak iliopsoas muscle, causing excessive anterior glide and pressure of the femoral head on anterior joint structures during flexion, including the capsule. Treatment approaches that incorporate muscle balance exercise can target these muscles and improve movement patterns.[18]

5. Effectiveness of other physiotherapy regimes

Research has shown that exercise is an effective treatment for many types of musculoskeletal pain[19,20], and has identified that exercise-based programmes can produce similar improvements in symptoms to surgery.[21] Personalised regimes of physiotherapy care have been effective and sometimes superior to surgery in managing musculoskeletal problems, with the advantage of significantly less risk than that associated with surgery.

Some examples include:

1. Knee arthroscopy used to be a routine treatment for patients with knee osteoarthritis. We now recognise after performing similar large scale randomised controlled trials that regimes of pain medication and physiotherapy-led exercise are more effective at managing a patients symptoms without the risks of surgery.[22,23]
2. Similar findings have been shown for the treatment of knee meniscal tears where exercise based physiotherapy is equally effective as surgery without the same level of risk.[24]
3. A large randomised trial of lumbar spine fusion versus intensive rehabilitation supervised by physiotherapist found no difference in outcome between groups but significantly less risk in the non-surgical treatment group.[25]

6. Define a protocol for best conventional care (comparator)

We performed a systematic review of non-operative care for FAI. This revealed little evidence of a standard for best conventional care, even though many NHS commissioners describe 'failure of conventional care' as a prerequisite for surgery.[26] There was some evidence that physiotherapy-led non-operative care is most frequently used.[11] This is complemented by established theory and evidence supporting treatment effects for physiotherapy in other painful musculoskeletal conditions including osteoarthritis and back pain.[27,28]

We used a combination of consensus methods (Delphi and Nominal Group techniques) among physiotherapists to agree a protocol for 'best conventional care'. We advertised to relevant networks of the Chartered Society of Physiotherapy (CSP) through their interactive communication system (iCSP) and in the *Frontline* magazine (twice monthly magazine posted to 52,000 CSP members in the UK). These advertisements invited physiotherapists to help develop a consensus for a best conventional care treatment protocol for FAI. Electronic invitations were also sent to physiotherapists in the United States and Australia known to us

through previous collaborative work on FAI. To encourage a process of 'snowball sampling' within the international community, these therapists were encouraged to invite colleagues with experience and interest in managing FAI to join in the consensus process.

We developed a physiotherapy-led, four component protocol, to be delivered over at least 12 weeks with a minimum of 6 one-to-one treatment contacts. It includes: (i) a detailed patient assessment; (ii) education and advice about FAI; (iii) help with pain relief including hip joint steroid injections; and (iv) an exercise programme that has the key features of individualisation, supervision and progression. We used a patient focus group to choose the most acceptable name for this protocol of best conventional care. The group made it clear that we should express that this was a coherent and valid alternative to surgery and different to physiotherapy likely to have been received already, and recommended the name Personalised Hip Therapy (PHT).

In the development of PHT we struck a balance between the need for a meaningful comparator for hip arthroscopy, the need to ensure PHT is different to previous physiotherapy that FAI patients may have experienced and the need for PHT to be deliverable in the NHS outside a trial. UK physiotherapists and patients felt that PHT was 'best' in that not all patients currently receive such a comprehensive package, but 'conventional' in that all its elements are widely used and the package is deliverable within usual constraints in the NHS.

7. Personalised Hip Therapy (PHT)

The personalised hip therapy programme is designed with 4 core components. Each patient should receive all four 4 core components over at least a 12 week programme. Optional additional components can be used where appropriate and additional symptoms that patients with femoroacetabular impingement (FAI) may present with can also be treated as per the treating physiotherapists preferred methods. All details of the programme and any additional interventions used must be recorded on the Case Report Form.

FOUR Core Components

7.1 Patient Education and advice

1. Education about FAI and available treatments
2. Advice about posture, gait and lifestyle behaviour modifications to try to avoid FAI. These may include:

    - Measures to encourage posterior pelvic tilt (reduce pelvic inclination)
    - Positioning when sitting, standing, sit to stand
    - Positioning when sleeping
    - Positioning when running / cycling where relevant

3. Advice about activities of daily living to try to avoid FAI (reducing / avoiding deep flexion, adduction and internal rotation of hip)
4. Advice about relative rest (for acute pain where patients cannot engage with their exercise-based personal hip programme) given that soft tissues take at least 8-10 weeks to heal. In particular, relative rest in a specific ROM where pain in that particular ROM is likely to represent ongoing impingement. Specific activity/sport technique advice and modification. Examples include running with a broader base to encourage abduction, cycling with less internal rotation on pedals, skiing with skis further apart and using knee flexion more than hip flexion to lower centre of gravity.

7.2 Patient Assessment

1. History: to include:

- History of presenting complaint
- Relieving and aggravating factors
- Past Medical History
- Medications
- Previous treatments tried
- Social History including occupation
- Patients concerns, fears and beliefs
- Patients individual requirements and expectations.

2. Examination

- Determine pain-free, passive ROM in the hip
- Determine the strength of motion in the hip in flexion, extension, abduction, adduction, internal and external rotation.
- Impingement test

7.3 Help with Pain Relief

1. Advice about anti-inflammatory medication for 2 to 4 weeks.
2. Advice about simple analgesics if they do not respond well to anti-inflammatory medication.
3. Engagement in, and adherence to, a personalised exercise programme

7.4 Exercise-based hip programme

1. An exercise programme that has the key features of individualisation, progression and supervision.
2. A phased exercise programme that begins with muscle control work, and progresses to stretching and strengthening with increasing ROM and resistance.
3. Muscle control / stability exercise (targeting pelvic and hip stabilisation, gluteal and abdominal muscles)
4. Strengthening / resistance exercise firstly in available range (pain-free ROM), and targets:
    - Gluteus maximus - extension
    - Short external rotators – external rotation
    - Gluteus medius – abduction
    - Abdominal muscles
    - Lower limb in general
5. Stretching exercise to improve hip external rotation and abduction in extension and flexion (but not vigorous stretching – no painful hard end stretches). Other muscles to be targeted if relevant for the patient include iliopsoas, hip flexors and rotators.
6. Exercise progression in terms of intensity and difficulty, gradually progressing to activity or sport-specific exercise where relevant.
7. A personalised and written exercise prescription that is progressed and revised over treatment sessions.
8. Encourage motivation and adherence through the use of a patient exercise diary to review progress.
9. Patients to have access to therabands, exercise balls and exercise mats.

8. Delivery of care

1. Care provided over at least 12 weeks
2. A minimum of 6 ‘contacts’ with the physiotherapist over 12 weeks
3. Ideally all 6 contacts are face-to-face but at least 3 should be face-to face, others can be via telephone/email support where that is needed due to geographical distance.
4. Further ‘booster’ follow-ups can be arranged between 12 weeks and 6 months
5. The maximum total number of contacts with physiotherapist is 10 including the optional further booster sessions.
6. Care provided by the same physiotherapist throughout where possible
7. Assessment between treatment sessions will be done by:
    - Subjective assessment
      Questions such as how do you currently rate your pain? Are your symptoms improving?
    - Objective assessment
      Pain levels using VAS
      Pain free ROM
      Exercise ability
    - Exercise adherence
      Review exercise diary and questions such as have you been able to complete the exercises you were given at the last visit?
8. Quality assurance
   A specific trial Case Report Form will need to be completed for each patient for each treatment contact, in order to accurately record all details of the interventions delivered to the patient.

9. Additional optional components

The following can be included in the patients care if the treating physiotherapist feels it is appropriate but must be recorded on the patients Case Report Form:

- ✓ **Manual Therapy**
  Hip joint mobilisations e.g. distraction, distraction with flexion, AP glides.
  Trigger point work
- ✓ **Hip Joint Injection**
  Potentially useful for patients who do not improve with 'core' treatment.
  Maximum of one steroid hip injection allowed.
- ✓ **Orthotics**
  Patients can be assessed for biomechanical abnormalities and either have these corrected by the treating physiotherapist. Alternatively patients can be referred to allied health care professionals such as a podiatrist for custom made insoles etc.
- ✓ **Taping**
  Taping techniques such as taping the thigh into external rotation and abduction to help with postural modification/reminding.
- ✓ **Group-based treatments**
  The core programme can be supplemented by but must **NOT** be substituted with group based treatment.
- ✓ **Treatment of additional pathology/symptoms**
  Physiotherapists are free to treat any additional pathology or symptoms that they feel is exacerbating a patient's FAI. Examples of this might include treating co-existing low back pain.

10. Protocol exclusions

- ✗ **Forceful manual techniques**
  Forceful manual techniques in restricted range of movement (Grade V mobilisations, or forceful stretching). No painful hard end stretches.
- ✗ **Student or technical instructor care**
  Care should not be delivered by a student or technical instructor
- ✗ **Hydrotherapy**
  Patients should not have hydrotherapy as part of their treatment
- ✗ **Acupuncture**
  Patients should not have acupuncture as part of their treatment
- ✗ **Electrotherapy**
  Patients should not have electrotherapy as part of their treatment

11. Comments and Suggestions

Please email the UK FASHIoN Team on ukfashion@warwick.ac.uk with any queries, comments or suggestions.

10. References

1. **Ganz R, Parvizi J, Beck M, Leunig M, Nötzli H, Siebenrock K.** Femoroacetabular impingement: a cause for osteoarthritis of the hip. *Clin Orthop Relat Res 2003-417:112-20.*
2. **Lavigne M, Parvizi J, Beck M, Siebenrock KA, Ganz R, Leunig M.** Anterior femoroacetabular impingement: part I. Techniques of joint preserving surgery. *Clin Orthop Relat Res 2004-418:61-6.*
3. **Ganz R, Leunig M, Leunig-Ganz K, Harris WH.** The etiology of osteoarthritis of the hip: an integrated mechanical concept. *Clin Orthop Relat Res 2008;466-2:264-72.*
4. **Allen D, Beaule PE, Ramadan O, Doucette S.** Prevalence of associated deformities and hip pain in patients with cam-type femoroacetabular impingement. *J Bone Joint Surg Br 2009;91-5:589-94.*
5. **Gosvig KK, Jacobsen S, Sonne-Holm S, Palm H, Troelsen A.** Prevalence of malformations of the hip joint and their relationship to sex,groin pain, and risk of osteoarthritis: a population-based survey. *J Bone Joint Surg Am 2010;92-5:1162-9.*
6. **Hack K, Di Primio G, Rakhra K, Beaule PE.** Prevalence of cam-type femoroacetabular impingement morphology in asymptomatic volunteers. *J Bone Joint Surg Am 2010;92-14:2436-44.*
7. **Takeyama A, Naito M, Shiramizu K, Kiyama T.** Prevalence of femoroacetabular impingement in Asian patients with osteoarthritis of the hip. *Int Orthop 2009;33-5:1229-32.*
8. **Clohisy JC, St John LC, Schutz AL.** Surgical treatment of femoroacetabular impingement: a systematic review of the literature. *Clin Orthop Relat Res 2010;468-2:555-64.*
9. **Botser IB, Smith TW, Jr., Nasser R, Domb BG.** Open surgical dislocation versus arthroscopy for femoroacetabular impingement: a comparison of clinical outcomes. *Arthroscopy 2011;27-2:270-8.*
10. **Matsuda DK, Carlisle JC, Arthurs SC, Wierks CH, Philippon MJ.** Comparative systematic review of the open dislocation, mini-open, and arthroscopic surgeries for femoroacetabular impingement. *Arthroscopy 2011;27-2:252-69.*
11. **Wall PDH, Fernandez M, Griffin DR, Foster NE.** Nonoperative Treatment for Femoroacetabular Impingement: A Systematic Review of the Literature. *PM R 2013;5-5:418-26.*
12. **Clohisy JC, Kim Y-J, Lurie J, Glyn-Jones S, Wall P, Wright R, Spindler K, Lohmander S.** Clinical Trials in Orthopaedics and the Future Direction of Clinical Investigations for Femoroacetabular Impingement. *Journal of the AAOS 2013;21-Supplement 1:47-52.*
13. **Ng VY, Arora N, Best TM, Pan X, Ellis TJ.** Efficacy of surgery for femoroacetabular impingement: a systematic review. *American Journal of Sports Medicine;38-11:2337-45.*
14. **Britton A, McKee M, Black N, McPherson K, Sanderson C, Bain C.** Choosing between randomised and non-randomised studies: a systematic review. *Health Technol Assess 1998;2-13:i-iv, 1-124.*
15. **Ross S, Grant A, Counsell C, Gillespie W, Russell I, Prescott R.** Barriers to participation in randomised controlled trials: a systematic review. *J Clin Epidemiol 1999;52-12:1143-56.*
16. ***Chakravertya LK, Snelling NJ**. Anterior hip pain – Have you considered femoroacetabular impingement? International Journal of Osteopathic Medicine 2012;15-1:22-7*

17. ***Kennedy MJ, Lamontagne M, Beaule PE**. Femoroacetabular impingement alters hip and pelvic biomechanics during gait Walking biomechanics of FAI. Gait Posture 2009;30-1:41-4*

18. Sahrmann SA. *Diagnosis and treatment of movement impairment syndromes. In:Mosby, 2002.*
19. National Institute for Health and Clinical Excellence*. The care and management of osteoarthritis in adults [CG59]. 2008.*
20. Frost H, Lamb SE, Robertson S. *A randomized controlled trial of exercise to improve mobility and function after elective knee arthroplasty. Feasibility, results and methodological difficulties. Clin Rehabil 202; 16-2:200-9*
21. Moseley JB, O'MalleyK, Petersen NJ, Menke TJ, Brody BA, Kuykendall DH, Hollingsworth JC, Ashton CM, Wray NP. *A controlled trial of arthroscopic surgery for osteoarthritits of the knee. N Engl J Med 2002;347-2:81-8*
22. Risberg MA.*Arthroscopic surgery provides no additional benefit over physiotherapy and medication for the treatment of knee osteoarthritis. Aust J Physiother 2009;55-2:137*
23. Herrlin S, Hallander M, Wange P, Weidenhielm L, Werner S. *Arthroscopic or conservative treatment of degenerative medial meniscal tears: a prospective randomised trial. Knee Surg Sports Traumatol Arthrosc 2007;15-4:393-401.*
24. Fairbank J, Frost H, Wildon-MacDonald J, Yu LM, Barker K, Collins R*. Randomised controlled trial to compare surgical stabilisation of the lumbar spine with an intensive rehabilitation programme for patients with chronic low back pain: the MRC spine stabilisation trial. BMJ 2005;330-7502:1233*
25.16. Ergina PL, Cook JA, Blazeby JM, Boutron I, Clavien PA, Reeves BC, Seiler CM, Balliol C, Altman DG, Aronson JK, Barkun JS, Campbell WB, Cook JA, Feldman LS, Flum DR, Glasziou P, Maddern GJ, Marshall JC, McCulloch P, Nicholl J, Strasberg SM, Meakins JL, Ashby D, Black N, Bunker J, Burton M, Campbell M, Chalkidou K, Chalmers I, de Leval M, Deeks J, Grant A, Gray M, Greenhalgh R, Jenicek M, Kehoe S, Lilford R, Littlejohns P, Loke Y, Madhock R, McPherson K, Rothwell P, Summerskill B, Taggart D, Tekkis P, Thompson M, Treasure T, Trohler U, Vandenbroucke J. Challenges in evaluating surgical innovation. *Lancet 2009;374-9695:1097-104.*
26.Cornwall and Isles of Scilly Primary Care Trust. Policy Statement: Open or Arthroscopic Surgery for the Treatment of Femoro-acetabular Impingement. 2011.
27. Fransen M, McConnell S. Exercise for osteoarthritis of the knee. *Cochrane Database Syst Rev 2008-4:CD004376.*
28. Hayden JA, van Tulder MW, Malmivaara AV, Koes BW. Meta-analysis: exercise therapy for nonspecific low back pain. *Ann Intern Med 2005;142-9:765-75*

FASHIoN

STUDYING HIP IMPINGEMENT
